# 41250 Machine Learning to Identify Predictors of Iatrogenic Injury Using Empirical Bayes Estimates: A Cohort Study of Pressure Injury Prevention

**DOI:** 10.1017/cts.2021.530

**Published:** 2021-03-30

**Authors:** William V. Padula, David G. Armstrong, Patricia M. Davidson

**Affiliations:** 1 University of Southern California; 2 Keck Medicine of USC; 3 Johns Hopkins School of Nursing

## Abstract

ABSTRACT IMPACT: A machine learning approach using electronic health records can combine descriptive, population-level factors of pressure injury outcomes. OBJECTIVES/GOALS: Pressure injuries cause 60,000 deaths and cost $26 billion annually in the US, but prevention is laborious. We used clinical data to develop a machine learning algorithm for predicting pressure injury risk and prescribe the timing of intervention to help clinicians balance competing priorities. METHODS/STUDY POPULATION: We obtained 94,745 electronic health records with 7,000 predictors to calibrate a predictive algorithm of pressure injury risk. Machine learning was used to mine features predicting changes in pressure injury risk; random forests outperformed neural networks, boosting and bagging in feature selection. These features were fit to multilevel ordered logistic regression to create an algorithm that generated empirical Bayes estimates informing a decision-rule for follow-up based on individual risk trajectories over time. We used cross-validation to verify predictive validity, and constrained optimization to select a best-fit algorithm that reduced the time required to trigger patient follow-up. RESULTS/ANTICIPATED RESULTS: The algorithm significantly improved prediction of pressure injury risk (p<0.001) with an area under the ROC curve of 0.60 compared to the Braden Scale, a traditional clinician instrument of pressure injury risk. At a specificity of 0.50, the model achieved a sensitivity of 0.63 within 2.5 patient-days. Machine learning identified categorical increases in risk when patients were prescribed vasopressors (OR=16.4, p<0.001), beta-blockers (OR=4.8, p<0.001), erythropoietin stimulating agents (OR=3.0, p<0.001), or were ordered a urinalysis screen (OR=9.1, p<0.001), lipid panel (OR=5.7, p<0.001) or pre-albumin panel (OR=2.0, p<0.001). DISCUSSION/SIGNIFICANCE OF FINDINGS: This algorithm could help hospitals conserve resources within a critical period of patient vulnerability for pressure injury not reimbursed by Medicare. Savings generated by this approach could justify investment in machine learning to develop electronic warning systems for many iatrogenic injuries.

